# Radical chain mechanism for the S_2_O_8_^2−^–S_2_O_3_^2−^–Cu(ii) flow system explains high-amplitude pH oscillations in the NH_4_OH-modified version†

**DOI:** 10.1039/d4ra02863e

**Published:** 2024-07-19

**Authors:** Krisztina Kurin-Csörgei, István Szalai, Miklós Orbán

**Affiliations:** a Laboratory of Nonlinear Chemical Dynamics, Institute of Chemistry, Eötvös University Pázmány Péter Sétány 1/A Budapest 1117 Hungary istvan.szalai@ttk.elte.hu

## Abstract

Peroxodisulfate is well known as an important reagent in analytical, environmental and other branches of chemistry, as well as in industrial processes. One of the most studied oxidative reactions peroxodisulfate participates in as an oxidizer is the Cu(ii)-catalyzed peroxodisulfate–thiosulfate reaction. When carried out in a flow reactor, this system shows oscillatory dynamics characterized by periodic changes in the Pt-potential and [O_2_] while it only displays variation in the pH with a few tenths of unit magnitude. Our recent experiments unveiled an increase of the amplitude of the pH oscillations that exceeds 4 units when NH_4_OH was introduced into the oscillatory flow system. The dynamics of Cu(ii)-catalyzed peroxodisulfate–thiosulfate reaction has been described in detail but the chemical mechanism explaining the oscillatory behavior has not been established. Based on what is known about the uncatalyzed reaction between peroxodisulfate and thiosulfate in the literature, we have developed a mechanism that includes radical chain reactions which can explain the oscillatory phenomena. The proposed mechanism includes 13 reactions with the radical ions SO_4_˙^−^, S_2_O_3_˙^−^, S_2_O_8_˙^−^, OH˙ and two acid–base equilibria, including the dissociation equilibrium of NH_4_OH accounting for its effect on the amplitude of pH oscillations. Using this model, we successfully simulated the behavior of this system: (1) the evolution of the concentrations of the initial reagents, radicals, and catalyst over time in batch configuration, (2) the periodic changes in the concentrations of radicals and the oxidized and reduced forms of the catalyst, pH and [O_2_] in flow conditions. Our model also explains the amplification of the pH cycles without impacting the redox processes when NH_4_OH is added, which is a novel phenomenon observed in nonlinear chemical reactions. The high amplitude pH oscillations we report in the peroxodisulfate–thiosulfate–Cu(ii)–NH_4_OH flow reaction may enable future applications where this system may serve (a) as a core oscillator in coupled chemical systems, or (b) as a pH oscillator capable of running in a closed reactor configuration.

## Abstract 2

Autocatalytic reaction of sulfate radical ions explains the oscillatory phenomena observed in flow reactors.

## Graphical abstract

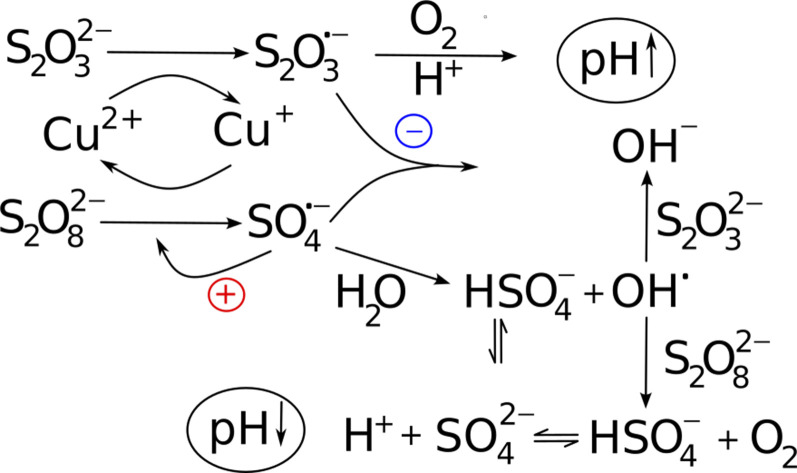

## 1 Introduction

The peroxodisulfate seems to be an ideal candidate to play the role of the oxidant in liquid phase oscillatory chemical reactions because the S_2_O_8_^2−^/SO_4_^2−^ pair has a very high redox potential and contains an element that possesses multiple oxidation states. Therefore, it is somewhat surprising that until now only two oscillatory reactions were discovered that utilize peroxodisulfate as the oxidizer, namely the Ag(i)-catalyzed reaction between peroxodisulfate and sulfide ions^1^ and the Cu(ii)-catalyzed oxidation of thiosulfate by peroxodisulfate ions.^2^ Detailed reports on the dynamical behavior of each system have been published, but comprehensive mechanisms that could explain the observations were not included in these studies. Our main goal was to develop a mechanism to account for the oscillations in the Cu(ii)-catalyzed system. The unique feature of this oscillator is that both the oxidant and the reductant are sulfur compounds. This implies that a mechanism capable of explaining the observed dynamics should be built up from sulfur species.

When the reaction between peroxodisulfate (S_2_O_8_^2−^) and thiosulfate (S_2_O_3_^2−^) was carried out in the presence of a trace amount of Cu(ii) ions in a continuous-flow stirred tank reactor (CSTR), the Pt electrode signal displayed oscillations with 50–100 mV amplitude. The periodic changes in the potential were accompanied by small amplitude oscillations in the pH (ΔpH ∼0.01–0.6 unit in the vicinity of pH ∼3) and in the concentration of dissolved oxygen.^2^ The observed dynamics is quite different from that of the seemingly analogous hydrogen peroxide–thiosulfate–Cu(ii) system, which shows large amplitude pH oscillations and is driven by hydrogen ion autocatalysis.^3^

Our recent experiments led to an unexpected discovery, reigniting our interest in the S_2_O_8_^2−^–S_2_O_3_^2−^–Cu^2+^ (PTCu) oscillator. We found that when NH_4_OH was introduced into the reactor along with the oscillatory mixture, it significantly increased the amplitude of pH oscillations, with ΔpH exceeding 4 units of magnitude. This unexpected expansion of the pH range into the alkaline pHs makes the NH_4_OH-modified oscillator a promising candidate to be used as the core oscillator in pH driven complex systems where oscillations are generated by coupling pH dependent equilibria to pH oscillations.

The S_2_O_8_^2−^–S_2_O_3_^2−^–Cu^2+^–NH_4_OH CSTR system (PTCuA) operates when the input [S_2_O_8_^2−^]_0_ to [S_2_O_3_^2−^]_0_ is in high stoichiometric excess in the reaction mixture. This unique feature allows us to make modifications to the experimental conditions in which oscillations may take place in semi-batch and batch-like configurations. Due to the simpler experimental setup the latter two could enable the design of practical applications relying on periodic changes of pH in closed containers.

This work summarizes the new experimental and mechanistic results we obtained recently in the original PTCu and the NH_4_OH-enhanced PTCuA systems. Our main goal was to establish a mechanism capable of explaining the dynamical behavior observed in the these systems as well as to demonstrate that the system can run in a semi-batch and batch-like experimental setup.

## 2 Methods

### 2.1 Experimental

The following chemicals were used: K_2_S_2_O_8_, Na_2_S_2_O_3_·5H_2_O, CuSO_4_·5H_2_O, NH_4_OH (Fisher Certified ACS), and sodium silicate solution (Sigma-Aldrich). Fresh stock solution of K_2_S_2_O_8_ (0.1 M) was prepared daily (it decomposes slowly at room temperature), and that of Na_2_S_2_O_3_ (0.01 M) could be used for several days. CuSO_4_ solution (0.001 M and 0.0001 M) was used, hydrolysis of the Cu^2+^ ions were prevented by setting the pH ∼4 using dilute H_2_SO_4_.

Three experimental configurations were used to study the behavior occurring when the stock solutions of the reactants S_2_O_8_^2−^, S_2_O_3_^2−^, Cu^2+^, and NH_4_OH were combined. Sustained oscillations were studied in a Continuously-fed Stirred Tank Reactor (CSTR): a 25 cm^3^ glass vessel equipped with a water jacket for thermal control (*T* = 25 °C) monitored by a Pt *vs.* Hg/Hg_2_SO_4_/K_2_SO_4_ electrode pair and a combined glass electrode (Orion); mixing was provided by a magnetic stirrer. The vessel had a Teflon cap with four openings for introducing the reactants through plastic tubes connected to a peristaltic pump. The excess reaction mixture was removed from the reactor at the overflow hole using a second pump. The Pt potential and the pH were measured with a pH meter (Consort). The analog signals were sampled every second and digitized with a 10 bit AD converter (DATAQ DI148-U). The pH *vs.* time data were stored and processed on a PC. In the semi-batch arrangement, a beaker of 100 cm^3^ served as a reactor, which contained the mixture of S_2_O_8_^2−^, Cu^2+^ and NH_4_OH; the stock solution of S_2_O_3_^2−^ was fed by a peristaltic pump at a very low rate. In order to produce a batch-like (closed) oscillatory system, all reagents must be in the reaction vessel. A beaker with a volume of 100 cm^3^ was used as a reactor that contained the mixture of S_2_O_8_^2−^, Cu^2+^ and NH_4_OH (V = 50 cm^3^); the continuous supply of Na_2_S_2_O_3_ was ensured by its dissolution from a silica gel layer coating the bottom of the beaker. This layer has been created by adding H_2_SO_4_ solution (3 cm^3^, 1.0 M) to dilute sodium silicate solution (8 cm^3^ of 2 : 1 mixture of water to waterglass) which also contained 1.19 mM Na_2_S_2_O_3_ (0.302 g), the silica gel forms within 1–2 minutes. The gel layer formed with a thickness of ∼5 mm, and a surface area of 12 cm^2^, it was rinsed with deionized water before use. To prevent the disruption of the silica gel layer stirring was carried out using a rotating propeller suspended from above the reactor.

### 2.2 Numerical methods

Integration of the systems of differential equations (see in ESI†) describing the kinetics of the system was carried out using variable-order method of the NumPy and SciPy packages.^4^ The applied relative and absolute tolerances were 10^−7^ and 10^−14^, respectively. The Python code used for the model presented in Table 1, the solve_ivp function with the BDF implicit multi-step simulations, is available under an Open Science Framework project.^5^ All initial (or input) feed concentrations were set to zero except those in which the values are explicitly mentioned. A flowchart representing the simulation method can be found in ESI (Fig. S1†).

## 3 Results

### 3.1 Experimental results

**Fig. 1:**
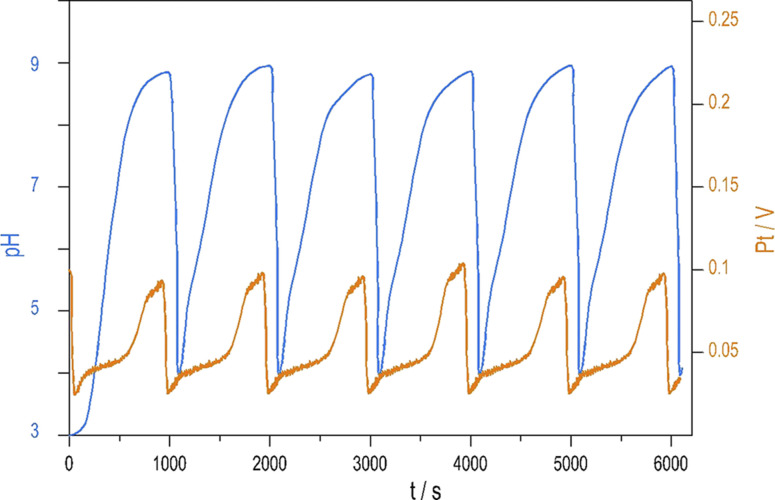
Oscillations in the pH (blue) and potential of the Pt electrode (red) were recorded in a CSTR using the optimum condition.

Introducing NH_4_OH to the PTCu oscillator significantly increases the amplitude of pH oscillations with minimal change to the oscillations measured by Pt electrode. The enhanced pH oscillations and the potential changes recorded in the PTCuA system are shown in Fig. 1. Our most frequently used parameters (optimum conditions) were as follows: [S_2_O_8_^2−^] = 0.02 M, [S_2_O_3_^2−^] = 0.005 M, [Cu^2+^] = 2 × 10^−5^ M, [NH_4_OH] = 0.005 M, *k*_0_ = 2 × 10^−3^ s^−1^, *T* = 25 °C. The amplitude and frequency of the pH peaks depend on the input concentration of the reagents and the flow rate (*k*_0_) (*e.g.*, ΔpH increases when [Cu^2+^] decreases from (5 × 10^−4^ M to 5 × 10^−6^ M) or when *k*_0_ is increased). We explored the behavior of the system, and provide here a combination of parameters where large amplitude pH oscillations appear in a CSTR (Fig. S2 and S3 in ESI†.)

**Fig. 2:**
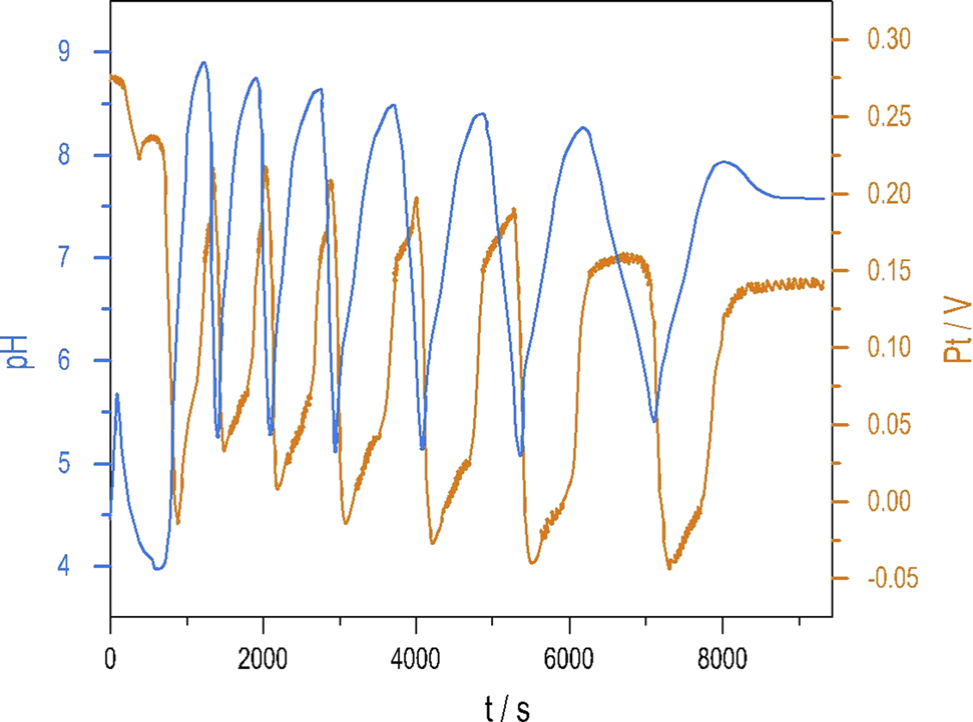
Oscillations in the pH (blue) and Pt potential (red) were measured in the S_2_O_8_^2−^–S_2_O_3_^2−^–Cu^2+^–NH_4_OH mixture under a semi-batch reactor arrangement. The concentrations in the reactor: [S_2_O_8_^2−^] = 0.021 M, [Cu^2+^] = 4 × 10^−5^ M [NH_4_OH] = 0.04 M; in the reservoir: [S_2_O_3_^2−^] = 0.0075 M.

To see oscillations in the Cu^2+^-catalyzed reaction between S_2_O_8_^2−^ and S_2_O_3_^2−^ either in the presence or absence of NH_4_OH, the oxidant should be applied in 5 to 10-fold stoichiometric excess to the reductant. This offers a way to run this system in semi-batch and closed reactor arrangements. To produce a semi-batch system, the solution of S_2_O_3_^2−^ was continuously introduced to the mixture of S_2_O_8_^2−^, NH_4_OH, and Cu^2+^ by use of a peristaltic pump, which operated with the appropriate rate. [NH_4_OH] = 0.04 M; in the reservoir: [S_2_O_3_^2−^] = 0.0075 M.The oscillations in the semi-batch arrangement are maintained until the concentration of the oxidant in the beaker becomes too low due to its consumption in the overall reaction and due to the dilution effect of the inflow of the reductant. Fig. 2 presents pH and potential oscillations lasting more than 2 h.

**Fig. 3:**
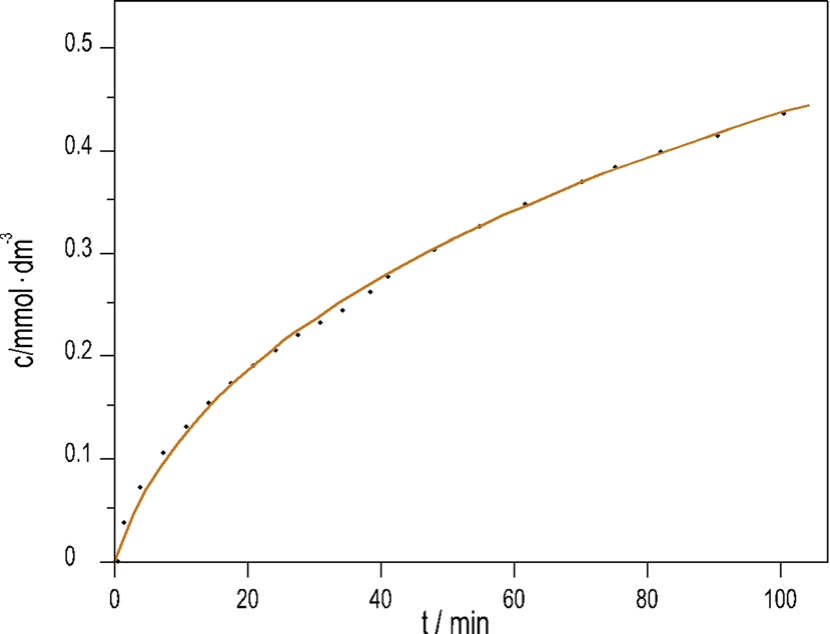
Dissolution of Na_2_S_2_O_3_ from silica gel layer into distilled water *vs.* time. (initial [Na_2_S_2_O_3_] = 1.19 mM; *T* = 25 °C).

We previously developed a method^6^ to produce pH oscillations in a batch-like configuration in other systems by replacing the inflow with a layer of silica gel impregnated with the key reactant; we applied it to the PTCuA system. The steady release rate of S_2_O_3_^2−^ from the gel layer is a critical parameter. The amount of S_2_O_3_^2−^ released into distilled water equivalent of the volume of the reaction mixture (50 cm^3^) was determined by iodometric titration at regular intervals. We established that S_2_O_3_^2−^ from the gel to the solution is released close to a constant rate over the course of 100 minutes, shown in Fig. 3.

**Fig. 4:**
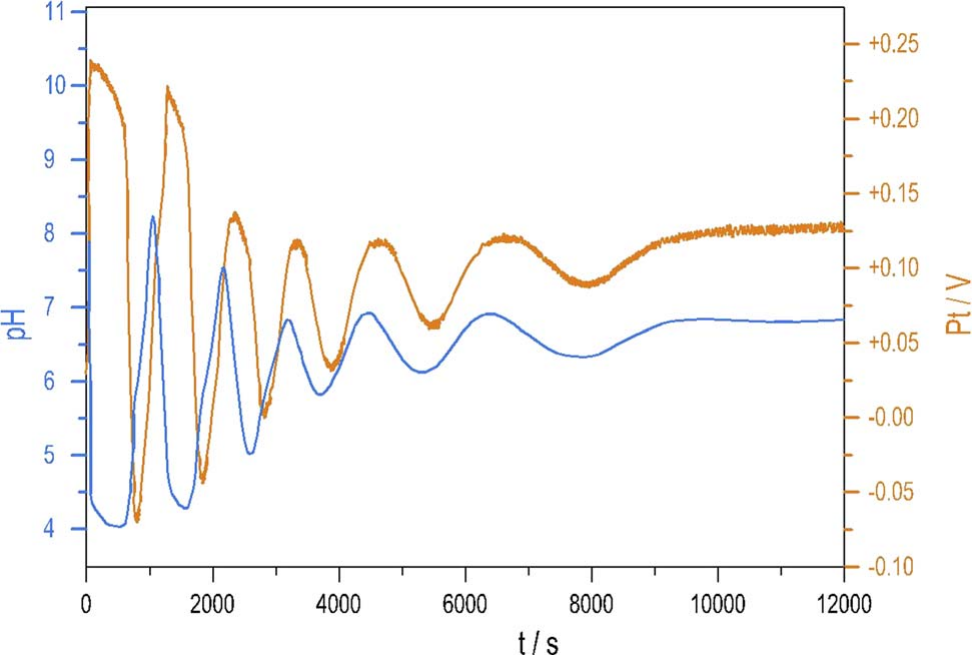
Oscillations in the pH (blue) and Pt potential (red) measured in the S_2_O_8_^2−^–S_2_O_3_^2−^–Cu^2+^–NH_4_OH mixture were measured under batch-like mode. Initial concentration in the reactor: [S_2_O_8_^2−^] = 0.021 M, [Cu^2+^] = 3 × 10^−5^ M, [NH_4_OH] = 0.05 M; [Na_2_S_2_O_3_] in the gel layer: 1.19 mM, *T* = 25 °C.

Fig. 4 shows the strongly damped oscillations in the pH and the potential of the Pt electrode recorded under a batch-like condition.

### 3.2 Numerical simulations

For the original S_2_O_8_^2−^–S_2_O_3_^2−^–Cu^2+^ oscillatory system we proposed a model that consisted of 15 steps in which neutral S_2_O_3_ and SO_4_ molecules as existing species, three radicals, S_2_O_3_˙^−^, SO_4_˙^−^, OH˙ were involved, the autocatalytic formation of S_2_O_3_˙^−^ and SO_4_˙^−^ radicals represented the (+) feedback. The mechanism could explain the potential oscillations, but oscillations in pH and [O_2_] were not considered.^2,7^ The mechanism has been criticized because of the presence of an illegal reaction loop for the formation of sulfate radicals.^8^ The new experimental observations presented here, especially the appearance of large amplitude pH oscillations in the presence of ammonia, prompted us to the revision of reaction mechanisms proposed earlier. We built a new model based on the widely accepted free radical chain mechanism of the peroxodisulfate–thiosulfate reaction suggested by Sorum and Edwards, which includes sulfate, thiosulfate, and hydroxyl radicals.^9^ This mechanism includes the following steps:

SE1 S_2_O_8_^2−^ → 2SO_4_˙^−^

SE2 SO_4_˙^−^ + H_2_O → HSO_4_^−^ + OH˙

SE3 S_2_O_3_^2−^ + OH˙ → OH^−^ + S_2_O_3_˙^−^

SE4 S_2_O_3_˙^−^ + S_2_O_8_^2−^ → S_2_O_3_SO_4_^2−^ + SO_4_˙^−^

SE5 S_2_O_3_˙^−^ + SO_4_˙^−^ → S_2_O_3_SO_4_^2−^

SE6 S_2_O_3_^2−^ + S_2_O_3_SO_4_^2−^ → S_4_O_6_^2−^ + SO_4_^2−^

SE7 S_2_O_3_^2−^ + O_2_ + 4H^+^ → 2S_4_O_6_^2−^ + 2H_2_O

We aimed to establish a mechanism that can explain the most relevant aspects of the oscillatory dynamics observed in experiments. In the construction of the model, several assumptions were made considering only the processes that are relevant under oscillatory conditions. We neglected the unimolecular decomposition of peroxodisulfate (SE1) and assumed that the catalyzed reaction (R3) is the primary pathway for its decomposition. We also neglected the complex formation reactions of the metal ions (copper(ii) and copper(i)), the pathway that leads to the formation of metallic copper and copper(i) sulfide,^10,11^ the formation of copper(iii), and the reactions between hydrogen peroxide and thiosulfate.^12^ We kept the hypothesis that the autocatalytic species is the sulfate radical, and therefore, unlike in the pH oscillators, the observed pH changes are only the consequence of the radical chain mechanism and not the driving force of the oscillations. In our model, presented in Table 1, 13 steps, three equilibrium, four radicals: S_2_O_3_˙^−^, SO_4_˙^−^, OH˙ and S_2_O_8_˙^−^ are involved. The existence of all species in each step is widely accepted in the works concerning the peroxodisulfate oxidation reactions. The rate constants were partly taken from references and have been estimated. In the simulations, the initial concentration of the reagents was similar to that used in the experiments.

**Table 1:** Mechanistic model of the peroxodisulfate–thiosulfate–copper(ii)–NH_4_OH oscillatory system

Reactions		Rate constants
R1	S_2_O_3_^2−^ + Cu^2+^ → S_2_O_3_˙^−^ + Cu^+^	*k*_1_ = 1 × 10^3^ M^−1^ s^−1^
R2	2S_2_O_3_˙^−^ → S_4_O_6_^2−^	*k*_2_ = 2 × 10^3^ M^−1^ s^−1^
R3	S_2_O_8_^2−^ + Cu^+^ → SO_4_˙^−^ + SO_4_^2−^ + Cu^2+^	*k*_3_ = 4 × 10^−1^ M^−1^ s^−1^
R4	2 SO_4_˙^−^ → S_2_O_8_^2−^	*k*_4_ = 8 × 10^8^ M^−1^ s^−1^
R5	SO_4_˙^−^ + H_2_O → HSO_4_^−^ + OH˙	*k*_5_ = 7 × 10^2^ M^−1^ s^−1^
R6	S_2_O_3_^2−^ + OH˙ → OH^−^ + S_2_O_3_˙^−^	*k*_6_ = 1 × 10^8^ M^−1^ s^−1^
R7	S_2_O_8_^2−^ + SO_4_˙^−^ → S_2_O_8_˙^−^ + SO_4_^2−^	*k*_7_ = 3.2 × 10^4^ M^−1^ s^−1^
R8	S_2_O_8_˙^−^ + H_2_O → 2 SO_4_˙^−^ + OH˙ + H^+^	*k*_8_ = 1 × 10^3^ M^−1^ s^−1^
R9	S_2_O_3_˙^−^ + O_2_ + H^+^ → products	*k*_9_ = 1 × 10^7^ M^−2^s^−1^
R10	2 OH˙ → H_2_O_2_	*k*_10_ = 1 × 10^8^ M^−1^ s^−1^
R11	S_2_O_8_^2−^ + H_2_O_2_ → O_2_ + 2 HSO_4_^−^	*k*_11_ = 1 × 10^5^ M^−1^ s^−1^
R12	S_2_O_3_˙^−^ + SO_4_˙^−^ → S_2_O_3_SO_4_^2−^	*k*_12_ = 2 × 10^6^ M^−1^ s^−1^
R13	S_2_O_3_^2−^ + S_2_O_3_SO_4_^2−^ → S_4_O_6_^2−^ + SO_4_^2−^	*k*_13_ = 1 × 10^5^ M^−1^ s^−1^
R14	HSO_4_^−^ ⇄ H^+^ + SO_4_^2–^	*k*_14_^f^ = 1.15 × 10^9^ s^−1^, *k*_14_^b^ = 1 × 10^11^ M^−1^ s^−1^
R15	H^+^ + OH^−^ ⇄ H_2_O	*k*_15_^f^ = 1.4 × 10^11^ M^−1^ s^−1^*k*_15_^b^ = 1.4 × 10^−3^ s^−1^
R16	NH_3_ + H_2_O ⇄ NH_4_^+^ + OH^−^	*k*_16_^f^ = 1.76 × 10^4^ s^−1^, *k*_16_^b^ = 1.0 × 10^9^ M^−1^ s^−1^

The oscillatory behavior of the PTCu and PTCuA reactions in batch and CSTR setups were simulated using the kinetic model in Table 1. The first two steps (R1 and R2) describe the fast reactions between thiosulfate and copper(ii). Reaction R1 is a simplified description of the reaction between thiosulfate and copper(ii), which starts with a rapid complex formation that is followed by a redox process:^10^

R1a Cu^2+^ + 2S_2_O_3_^2−^ ⇄ Cu(S_2_O_3_)_2_^2−^

R1b 2Cu(S_2_O_3_)_2_^2−^ → 2CuS_2_O_3_^−^ + S_4_O_6_^2−^

Reaction R3 describes the catalytic effect of Cu(i), and R4 is the recombination of sulfate radicals.^13^ The model includes the reactions (R5, R6, R9, R12, and R13) proposed by Sorum and Edwards for the uncatalyzed peroxodisulfate–thiosulfate reaction.^9^ The autocatalytic formation of sulfate radicals in reactions R7 and R8 is suggested to proceed through the formation of S_2_O_8_˙^−^. Reaction R7 was introduced to explain the decay of SO_4_˙^−^ in aqueous solution and is often used in models related to peroxodisulfate oxidations.^14–16^ We also propose that S_2_O_8_˙^−^ hydrolysis in reaction R8 produces two sulfate radicals. We do not have direct evidence for reaction R8, but this assumption introduces the autocatalytic cycle necessary to develop oscillatory dynamics. The decay of OH˙ in reaction R10 results in the formation of hydrogen peroxide that reacts with peroxodisulfate in reaction R11.^13^ The last three steps (R14, R15, and R16) in the model are the relevant acid–base equilibria.

**Fig. 5:**
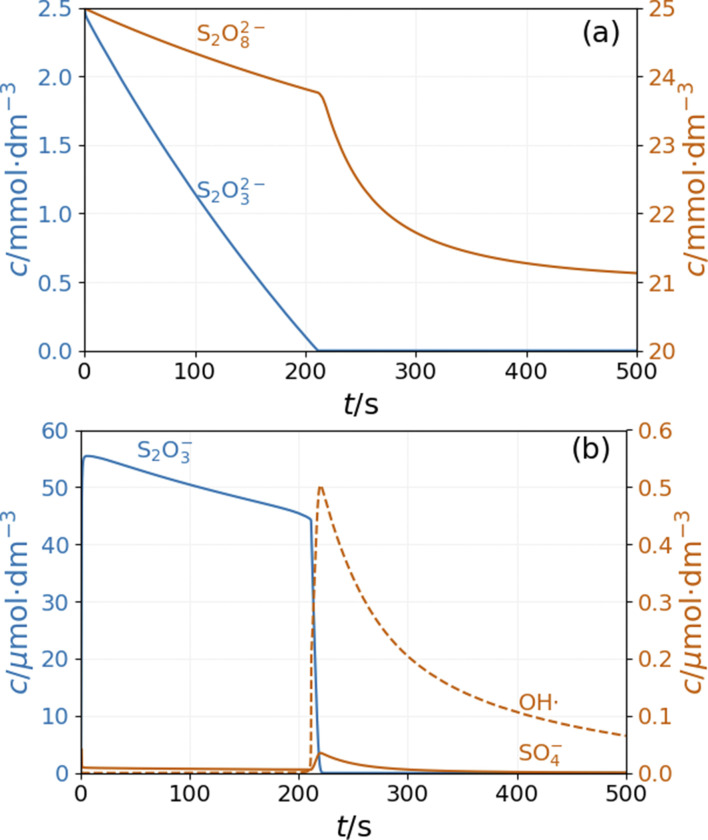
Simulation of the batch dynamics of the peroxodisulfate–thiosulfate–copper(ii) reaction: concentration *vs*. time curves of (a) S_2_O_8_^2−^ and S_2_O_3_^2−^ and (b) SO_4_˙^−^, S_2_O_3_˙^−^ and OH˙ The parameters used in the simulations are: [S_2_O_3_^2−^]_0_ = 0.0025 M, [S_2_O_8_^2−^]_0_ = 0.025 M, [Cu^2+^]_0_ = 2.5 × 10^−5^ M, [H^+^]_0_ = 10^−5^ M, [O_2_]_0_ = 2 × 10^−4^ M.

In the simulations of batch reaction, the temporal behavior of the model shows two distinct sections. In the first part, until 200 s of the simulation presented in Fig. 5, reactions R1 and R3 result in a monotonous decrease of peroxodisulfate and thiosulfate. The relatively large concentration of S_2_O_3_˙^−^ radical suppresses the autocatalytic formation of SO_4_˙^−^ radical by reactions R12 and R13. This period is represented by high pH and high Cu^+^ concentration (Fig. S4 and S5 in ESI†). When the thiosulfate is almost completely consumed, the autocatalytic process represented by reactions R7 and R8 produces a sharp increase in the concentrations of SO_4_˙^−^ and OH˙ radicals. The decay of these radicals is caused by reactions R5 and R10. The pH and the concentration of Cu^+^ decrease in this latter part of the reaction (Fig. S4 and S5 in ESI†). In agreement with the experimental observations, the simulations show that excess peroxodisulfate favors sulfate radical autocatalysis.^2^ Only when thiosulfate is completely consumed by the excess peroxodisulfate can the sulfate and hydroxyl radicals start to accumulate in the rapid autocatalytic process.

**Fig. 6:**
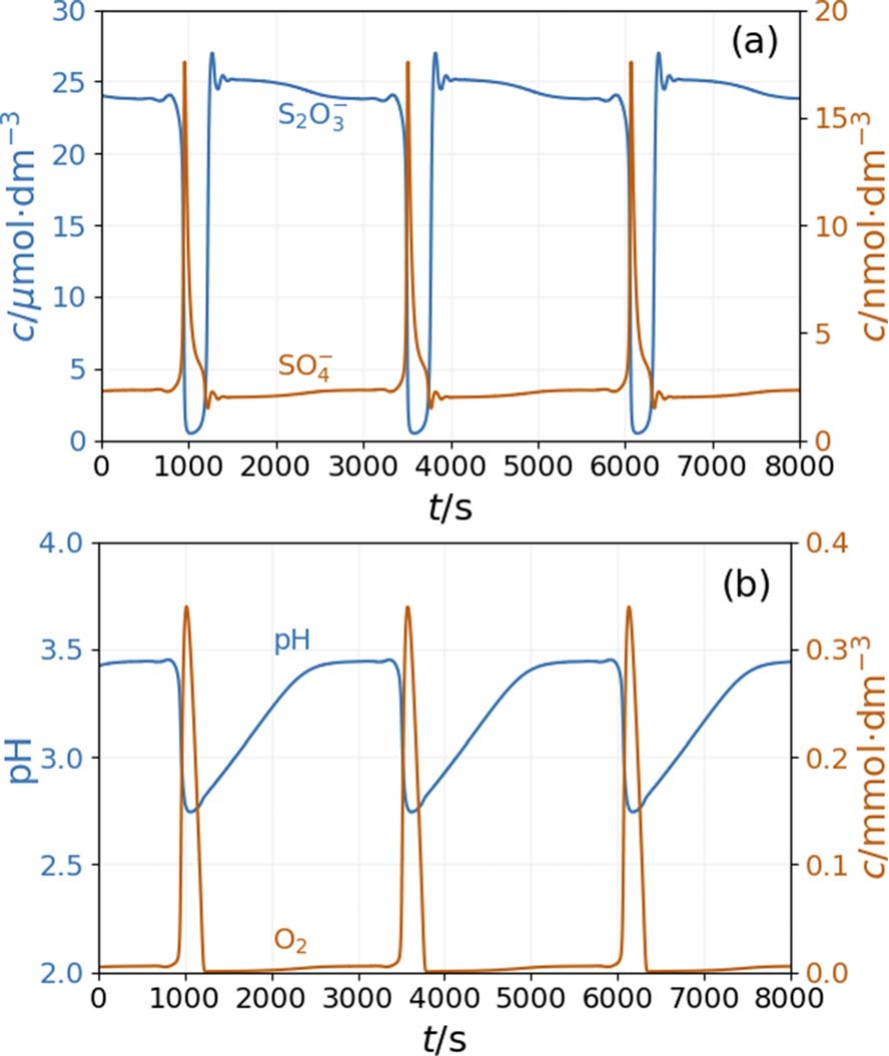
Simulation of the CSTR dynamics of the peroxodisulfate–thiosulfate–copper(ii) reaction: oscillations in the (a) concentrations of S_2_O_3_˙^−^ and SO_4_˙^−^ radicals and (b) pH and concentration of O_2_. The parameters used in the simulations are: [S_2_O_3_^2−^]_0_ = 0.0025 M, [S_2_O_8_^2−^]_0_ = 0.025 M, [Cu^2+^]_0_ = 2.5 × 10^−5^ M, [H^+^]_0_ = 10^−5^ M, [O_2_]_0_ = 2 × 10^−4^ M, *k*_0_ = 1.2 × 10^−3^ s^−1^.

The model shows sustained oscillations under CSTR conditions. Fig. 6(a) presents out of phase oscillations in the concentration of SO_4_˙^−^ and S_2_O_3_˙^−^ radical consistent with the observations in batch results. Two distinct parts of an oscillatory cycle can be recognized The longer part is characterized by a high concentration of S_2_O_3_˙^−^ and a low concentration of SO_4_˙^−^. When the concentration of S_2_O_3_^˙−^ decreases below a critical value, the autocatalytic formation of SO_4_˙^−^ results in a sharp peak in the concentration of O_2_ and a decrease in the pH {Fig. 6(b)}. The sources of oxygen are the inflow (the amount corresponds to the oxygen dissolved from the air into the input solutions) and reaction R11, while at high concentrations of S_2_O_3_˙^−^ radicals, oxygen is consumed by reaction R9. The increase in concentration of SO_4_˙^−^ results in a pH drop due to reactions R5 and R8. The hydrogen ions are removed by reactions R6 and R9 and the outflow. The concentration oscillations of the copper species are shown in Fig S6 and S7 in ESI.†

**Fig. 7:**
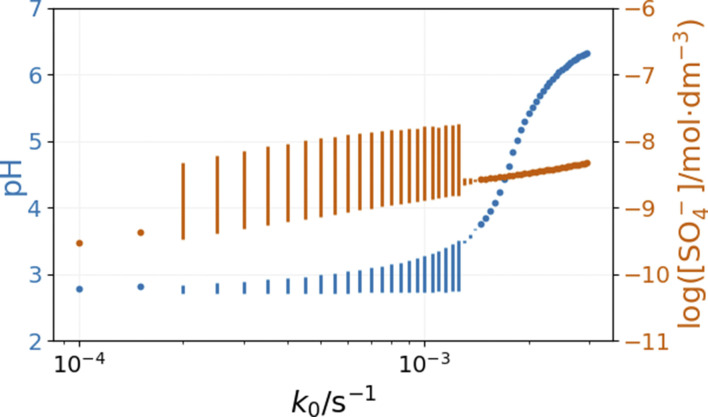
Simulated bifurcation diagram of the peroxodisulfate–thiosulfate–copper(ii) reaction. The dots correspond to stationary states, and the vertical lines indicate the amplitude of oscillation. The parameters used in the simulations are: [S_2_O_3_^2−^]_0_ = 0.0025 M, [S_2_O_8_^2−^]_0_ = 0.025 M, [Cu^2+^]_0_ = 2.5 × 10^−5^ M, [H^+^]_0_ = 10^−5^ M, [O_2_]_0_ = 2 × 10^−4^ M.

The inflow of fresh chemicals, especially thiosulfate, and the outflow of products are in a delicate balance with the reactions that consume and produce intermediates when oscillations occur. The simulated bifurcation diagram in Fig. 7 uncovers the CSTR dynamics as a function of *k*_0_, which is the reciprocal residence time in the CSTR. At large values of *k*_0_, above 2 × 10^−3^ s^−1^, the stationary state of the CSTR mixture is characterized by a pH of around 6.

The composition of the CSTR mixture is close to that in the 200 s of the batch simulations in Fig. 5. The relatively fast inflow of thiosulfate, compared to its overall consumption rate by peroxodisulfate, keeps the concentration of S_2_O_3_˙^−^ radicals high, which results in a high pH. As *k*_0_ decreases (the residence time increases), the pH and concentration of SO_4_˙^−^ of the corresponding stationary states decrease. The oscillatory phenomenon is found in the range of *k*_0_ = 2 × 10^−4^ and 1.35 × 10^−3^ s^−1^. The oscillations appear at *k*_0_ = 2 × 10^−4^ s^−1^ with a finite amplitude, a sign of a subcritical bifurcation. However, at 1.35 × 10^−3^ s^−1^, the oscillations vanish, which is typical for a supercritical Hopf bifurcation. The amplitude of the pH oscillations decreases dramatically with the decrease of *k*_0_ as the contribution of the outflow to the depletion of H^+^ concentration decreases as well. The strong effect of the flow rate on the amplitude of pH oscillations is essential in explaining the effects of ammonia.

**Fig. 8:**
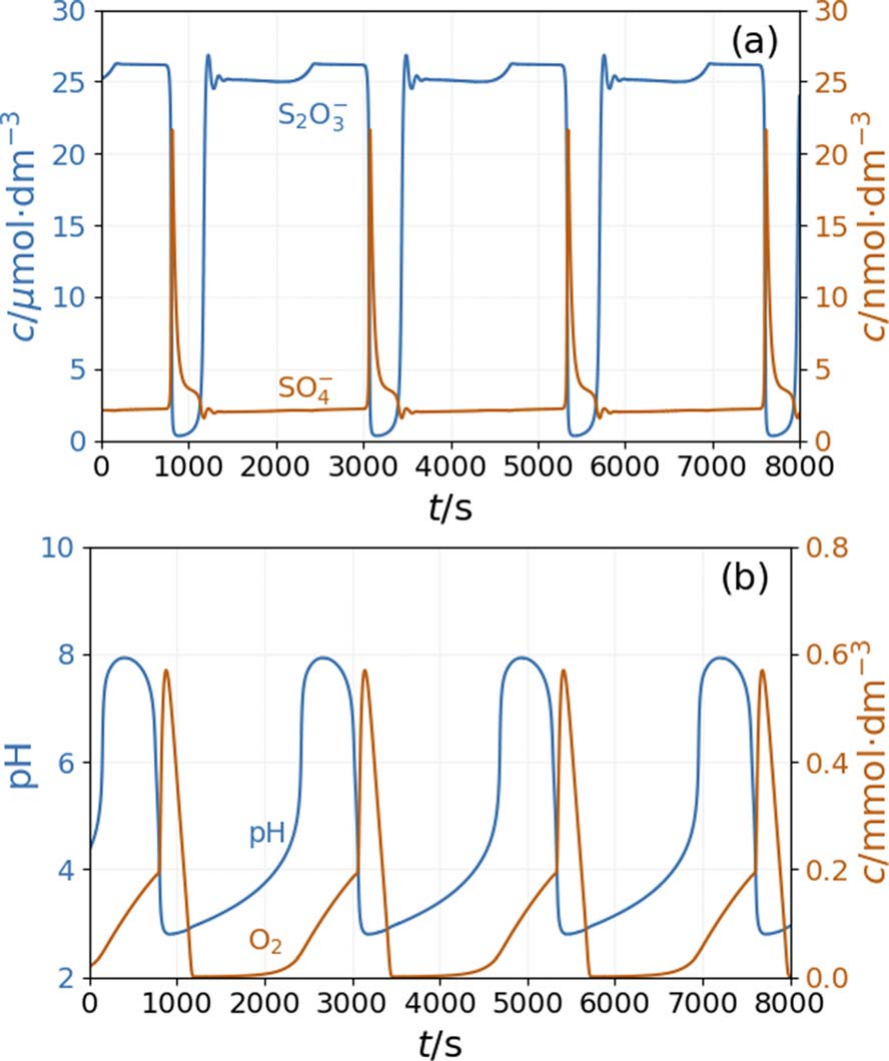
Simulation of the CSTR dynamics of the peroxodisulfate–thiosulfate–copper(ii) reaction in the presence of ammonia: simulation of the CSTR dynamics of the peroxodisulfate–thiosulfate–copper(ii) reaction: oscillations in the (a) concentrations of S_2_O_3_˙^−^ and SO_4_˙^−^ radicals and (b) pH and concentration of O_2_. The parameters used in the simulations are: [S_2_O_3_^2−^]_0_ = 0.0025 M, [S_2_O_8_^2−^]_0_ = 0.025 M, [Cu^2+^]_0_ = 2.5 × 10^−5^ M, [H^+^]_0_ = 10^−5^ M, [NH_3_]_0_ = 5 × 10^−4^ M, [O_2_]_0_ = 2 × 10^−4^ M, *k*_0_ = 1.2 × 10^−3^ s^−1^.

In our initial approach we introduced only the R16 acid–base equilibria to model the effect of ammonia on the system's dynamics observed in the experiments. The simulations reveal that in a specific range of input feed concentrations of ammonia, the pH amplitude of the oscillations increases dramatically {Fig. 8(b)}. At the same time, the amplitude of oscillations in [O_2_] increases by a factor of two. However, the amplitude of the oscillations of SO_4_˙^−^ and S_2_O_3_˙^−^ radicals {Fig. 8(a)} and the copper species (Fig. S6 in ESI†) changes only slightly.

**Fig. 9:**
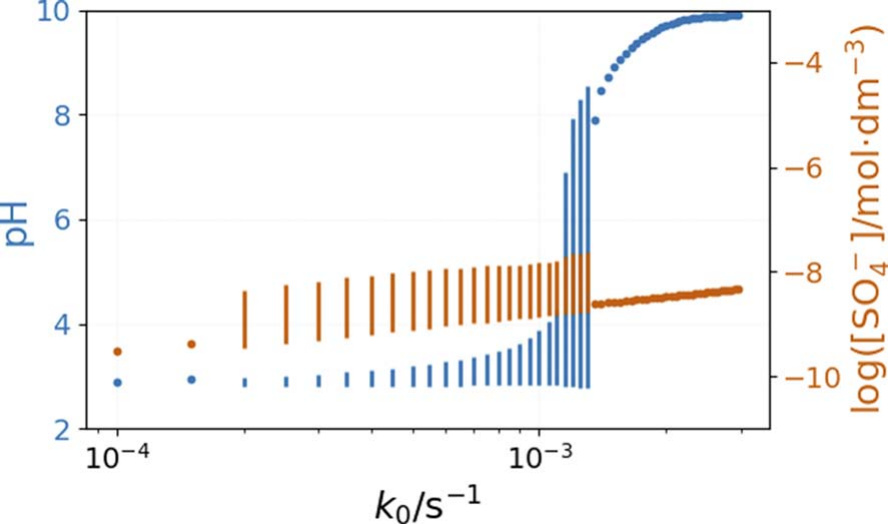
Simulated bifurcation diagram of the peroxodisulfate–thiosulfate–copper(ii) reaction in the presence of ammonia. The dots correspond to stationary states, and the vertical lines indicate the amplitude of oscillation. The parameters used in the simulations are: [S_2_O_3_^2−^]_0_ = 0.0025 M, [S_2_O_8_^2−^]_0_ = 0.025 M, [Cu^2+^]_0_ = 2.5 × 10^−5^ M, [NH_3_]_0_ = 5 × 10^−4^ M, [H^+^]_0_ = 10^−5^ M, [O_2_]_0_ = 2 × 10^−4^ M.

**Fig. 10:**
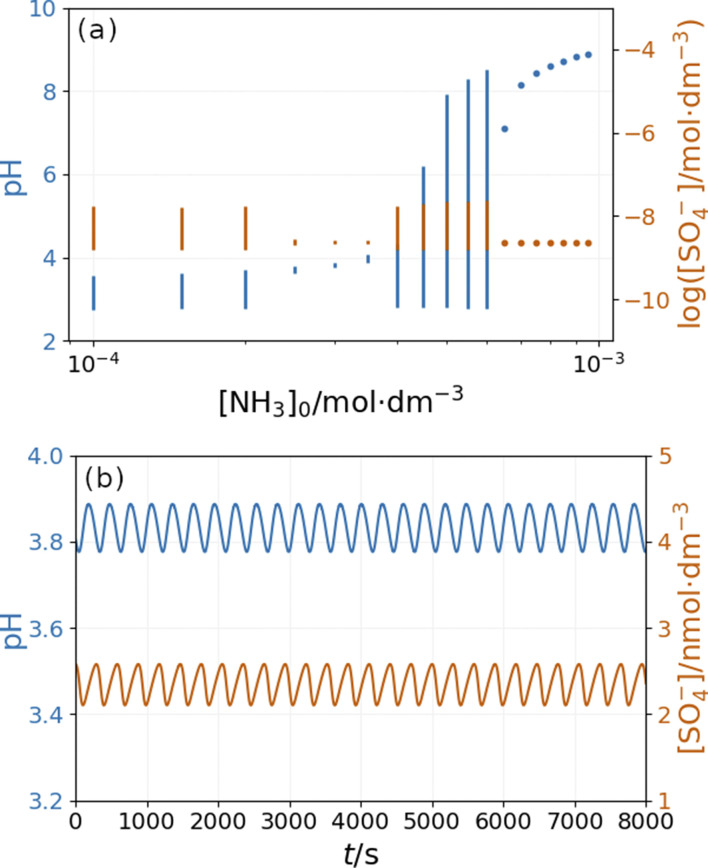
Simulated bifurcation diagram of the peroxodisulfate–thiosulfate–copper(ii) reaction in the presence of ammonia at *k*_0_ = 1.2 × 10^−3^ s^−1^ (a) and and oscillations at [NH_3_]_0_ = 3 × 10^−3^ M (b). The dots correspond to stationary states and the vertical lines indicate the amplitude of oscillation. The parameters used in the simulations are: [S_2_O_3_^2−^]_0_ = 0.0025 M, [S_2_O_8_^2−^]_0_ = 0.025 M, [Cu^2+^]_0_ = 2.5 × 10^−5^ M, [H^+^]_0_ = 10^−5^ M, [O_2_]_0_ = 2 × 10^−4^ M.

The bifurcation diagrams in Fig. 9 and 10 show the effect of flowrate (*k*_0_) and the input [NH_4_OH] on the amplitude of pH oscillations simulated in the PTCuA flow system. Fig. 9 indicates that the effect of ammonia on the amplitude of pH oscillation depends on the actual value of *k*_0_ and it is most prominent in the neighborhood of the bifurcation point. It is notable that the presence of ammonia just slightly affects the oscillations in the concentration of the autocatalytic species [SO_4_˙^−^]. As we discussed previously, the rate at which hydrogen ions are washed out strongly influences the amplitude of pH oscillations. Now, the presence of ammonia in the input flow enhances the depletion of hydrogen ions, and the pH increases independently of the presence of thiosulfate and thiosulfate radicals.

Fig. 10 depicts the simulated states *vs.* the input [NH_4_OH] under similar conditions as in Fig. 9. The presence of ammonia amplifies the pH changes during the oscillations above a critical value (4 × 10^−4^ M in Fig. 10) of its input feed concentrations. However, below this critical input feed concentration of ammonia, the amplitude of oscillations is reduced due to the decrease in the rate of reaction R9. This oscillatory mode is characterized by a very low amplitude and a high frequency {Fig. 10(b)}. Simulations performed in the absence of ammonia but at different flow rates reveal that this type of oscillation can be observed at a lower rate of reaction R9 (Fig. S9 in ESI†). At very low input feed concentrations (below 2 × 10^−4^ M in Fig. 10), the effect of ammonia becomes negligible.

Additional simulations performed in the absence of ammonia but at different input feed pHs show that the appearance of the high-amplitude pH oscillations is caused by the alkalinity of the input feed (Fig. S10 in ESI†).

## 4 Discussion

The overall stoichiometry of the reaction between S_2_O_8_^2−^ and S_2_O_3_^2−^, either in the absence or presence of Cu^2+^ and NH_4_OH, is written in eqn (1):^17^

1 S_2_O_8_^2−^ + 2S_2_O_3_^2−^ → S_4_O_6_^2−^ + 2SO_4_^2−^

This stoichiometry implies that H^+^ and OH^−^ ions are not involved directly in this reaction, therefore appearance of pH oscillations is not expected when the two react. However, in an open configuration the PTCu reaction displays small amplitude oscillations in the [H^+^], which increase considerably when NH_4_OH was introduced to the reaction feedstock. The exact role of the ammonia is not clear. It is a weak base (*p*Ks = 9.3), and due to its alkaline character, it may contribute to the increase of the pH amplitude, but its basicity alone does not induce large pH peaks. Substitution of NH_4_OH with an equivalent amount of NaOH resulted in only small amplitude or no oscillations. The model was extended with the dissociation equilibrium of the ammonia to consider the effect of the NH_4_OH on the dynamics of the PTCu system. Using the extended mechanism, we successfully simulated the small amplitude oscillations observed in the PTCu flow system and the largely amplified pH peaks measured in the PTCuA reaction. The significant difference in the pH amplitudes is seen when Fig. 6(b) is compared to Fig. 8(b). These two figures also show the simulated periodic changes in the [O_2_]. In this oscillatory cycle, the oxygen is taken up from the air or forms in step R9 and consumed in step R7.

The chemical role of NH_4_OH in generating large pH changes in the PTCuA flow system needs further explanation, *e.g.*, by including ammonia oxidation by peroxodisulfate. However, this work has established that under specific conditions (a narrow range of input [NH_4_OH] and flow rate), high-amplitude pH oscillations were observed experimentally and successfully simulated with a suggested model. According to our model, ammonia has a dual role, as it increases the pH of the input feed solution and affects the rate of the pH-dependent rate of reaction R9. It is also essential to notice, that there is no significant change in the oscillations of sulfate radicals when the amplitude of pH oscillations increases due to ammonia. These findings are significant in advancing our understanding of the complex dynamics of chemical reactions.

## 5 Conclusions

We developed a mechanism that accounts for the oscillatory kinetics in the PTCu and PTCuA flow systems. The uniqueness of this model is that it consists almost entirely of radical steps. Among the known liquid phase oscillatory reactions, no other system that operates according to the radical chain mechanism has been reported so far. We anticipate that the autocatalytic formation of SO_4_˙^−^ by reactions R7 and R8 might play a significant role in the mechanism of the Ag^+^-ion catalyzed S_2_O_8_^2−^–S^2−^ oscillatory reaction.^1^ The other outcome of this work is the observation of induced pH amplification in the PTCuA reaction. This result can be viewed as a new phenomenon among the dynamical behaviors occurring in nonlinear chemical systems. Despite displaying large pH changes as shown in Fig. 1, this system does not belong to the family of pH driven oscillators in which the driving force for the pH oscillations is the H^+^ autocatalysis,^18^ this is not present in the PTCuA reaction. This result demonstrates that large amplitude pH oscillations can evolve even if there is no H^+^ autocatalysis in the mechanism. The semi-batch and batch-like mode operation is advantageous in potential applications where the autonomous pH signal drives the changes between free or complexed forms of non-redox species or the motion of a pH-responsive coupled system.^19,20^

## Data availability

The code for the numerical simulation can be found at https://osf.io/7nxkg/ with DOI https://doi.org/10.17605/OSF.IO/7NXKG

## Conflicts of interest

There are no conflicts to declare.

## Supplementary Material

RA-014-D4RA02863E-s001

## References

[cit1] Quyang Q., De Kepper P. (1987). J. Phys. Chem..

[cit2] Orbán M., Epstein I. R. (1989). J. Am. Chem. Soc..

[cit3] Orbán M., Epstein I. R. (1987). J. Am. Chem. Soc..

[cit4] Harris C. R., Millman K. J., Van Der Walt S. J., Gommers R., Virtanen P., Cournapeau D., Oliphant T. E. (2020). Nature.

[cit5] Szalai I. , Peroxodisulfate–Thiosulfate–Cu(ii) Mechanism, 2024, March 6, Retrieved from https://osf.io/7nxkg/

[cit6] Poros E., Horváth V., Kurin-Csörgei K., Epstein I. R., Orbán M. (2011). J. Am. Chem. Soc..

[cit7] Orbán M., Kurin-Csörgei K., Rábai G., Epstein I. R. (2000). Chem. Eng. Sci..

[cit8] Stanbury D. M., Hoffman D. (2019). J. Phys. Chem. A.

[cit9] Sorum C. H., Edwards J. O. (1952). J. Am. Chem. Soc..

[cit10] Rabai G., Epstein I. R. (1992). Inorg. Chem..

[cit11] Unguresan M. L., Niac G. (2007). Bioinorg. React. Mech..

[cit12] Kurin-Csörgei K., Orbán M., Rábai G., Epstein I. R. (1996). J. Chem. Soc., Faraday Trans..

[cit13] House D. A. (1962). Chem. Rev..

[cit14] McElroy W. J., Waygood S. J. (1990). J. Chem. Soc., Faraday Trans..

[cit15] Chitose N., Katsumura Y., Domae M., Zuo Z., Murakami T. (1999). Radiat. Phys. Chem..

[cit16] Wojnárovits L., Takács E. (2019). Chemosphere.

[cit17] Byerley J. J., Fouda A. A., Rempel G. L. (1973). J. Chem. Soc., Faraday Trans..

[cit18] Ouyang Q., De Kepper P. (1987). J. Phys. Chem..

[cit19] Dúzs B., Lagzi I., Szalai I. (2023). ChemSystemsChem.

[cit20] Sharma C., Maity I., Walther A. (2023). Chem. Commun..

