# Supplementary material for: Radical chain mechanism for the S2O82−–S2O32−–Cu(ii) flow system explains high-amplitude pH oscillations in the NH4OH-modified version
Source: RSC Adv. 2024 Jul 19;14(32):22939–46. doi: 10.1039/d4ra02863e (PMC11258961; doi:10.1039/d4ra02863e)
Supplement: RA-014-D4RA02863E-s001 [file RA-014-D4RA02863E-s001.pdf]

Electronic Supplementary Material (ESI) for RSC Advances.
This journal is © The Royal Society of Chemistry 2024

Electronic Supplementary Information

Radical chain mechanism for the $S_2O_8^{2-}$ – $S_2O_3^{2-}$ – Cu(II) flow system explains high-amplitude pH oscillations in the NH_4OH-modified version

Krisztina Kurin-Csörgei, István Szalai and Miklós Orbán
Laboratory of Nonlinear Chemical Dynamics, Institute of Chemistry, Eötvös University,
Pázmány Péter sétány 1/A, Budapest 1117, Hungary

Derivation of the kinetic differential equations

Rate laws:

$v_1=k_1[S_2O_3^{2-}][Cu^{2+}]$
$v_2=k_2[S_2O_3^{-}]^2$
$v_3=k_3[S_2O_8^{2-}][Cu^{+}]$
$v_4=k_4[SO_4^{-}]^2$
$v_5=k_5[SO_4^{-}]$
$v_6=k_6[S_2O_3^{2-}][OH\cdot]$
$v_7=k_7[S_2O_8^{2-}][SO_4^{-}]$
$v_8=k_8[S_2O_8^{-}]$
$v_9=k_9[S_2O_3^{-}][O_2][H^+]$
$v_{10}=k_{10}[OH\cdot]^2$
$v_{11}=k_{11}[S_2O_8^{2-}][H_2O_2]$
$v_{12}=k_{12}[S_2O_3^{-}][SO_4^{-}]$
$v_{13}=k_{13}[S_2O_3^{2-}][S_2O_3SO_4^{2-}]$
$v_{14}=k^f_{14}[HSO_4^{-}]- k^b_{14}[H^+][SO_4^{2-}]$
$v_{15}=k^f_{15}[H^+][OH^-]- k^b_{15}$
$v_{16}=k^f_{16}[NH_3]- k^b_{16}[NH_4^+][OH^-]$

Differential equations for the simulations of the CSTR behavior:

$d[S_2O_3^{2-}]/dt=-v_1-v_6-v_{13}+k_0([S_2O_3^{2-}]_0-[S_2O_3^{2-}])$
$d[Cu^{2+}]/dt=-v_1+v_3+k_0([Cu^{2+}]_0-[Cu^{2+}])$
$d[S_2O_3^{-}]/dt=v_1-2v_2+v_6-v_9-v_{12}+k_0([S_2O_3^{-}]_0-[S_2O_3^{-}])$
$d[Cu^+]/dt=v_1-v_3+k_0([Cu^+]_0-[Cu^+])$
$d[S_4O_6^{2-}]/dt=v_2+v_{13}+k_0([S_4O_6^{2-}]_0-[S_4O_6^{2-}])$
$d[S_2O_8^{2-}]/dt=-v_3+v_4-v_7-v_{10}+k_0([S_2O_8^{2-}]_0-[S_2O_8^{2-}])$
$d[SO_4^{-}]/dt=v_3-2v_4-v_5-v_7+2v_8-v_{12}+k_0([SO_4^{-}]_0-[SO_4^{-}])$
$d[SO_4^{2-}]/dt=v_3+v_{14}+v_7+v_{13}+k_0([SO_4^{2-}]_0-[SO_4^{2-}])$
$d[HSO_4^{-}]/dt=-v_{14}+v_5+2v_{11}+k_0([HSO_4^{-}]_0-[HSO_4^{-}])$
$d[OH\cdot]/dt= v_5-v_6+v_8-2v_{10}+k_0([OH\cdot]_0-[OH\cdot])$
$d[H^+]/dt=--v_{15}+v_{14}-v_9+v_8+k_0([H^+]_0-[H^+])$
$d[OH^-]/dt=-v_{15}+v_6+v_{16}+k_0([OH^-]_0-[OH^-])$
$d[S_2O_8^{-}]/dt=v_7-v_8+k_0([S_2O_8^{-}]_0-[S_2O_8^{-}])$
$d[O_2]/dt=-v_9+v_{11}+k_0([O_2]_0-[O_2])$
$d[H_2O_2]/dt=v_{10}-v_{11}+k_0([H_2O_2]_0-[H_2O_2])$
$d[S_2O_3SO_4^{2-}]/dt=v_{12}-v_{13}+k_0([S_2O_3SO_4^{2-}]_0-[S_2O_3SO_4^{2-}])$
$d[HN_3]/dt=-v_{16}+k_0([HN_3]_0-[HN_3])$
$d[HN_4^+]/dt=v_{16}+k_0([HN_4^+]_0-[HN_4^+])$

Supplementary Figures

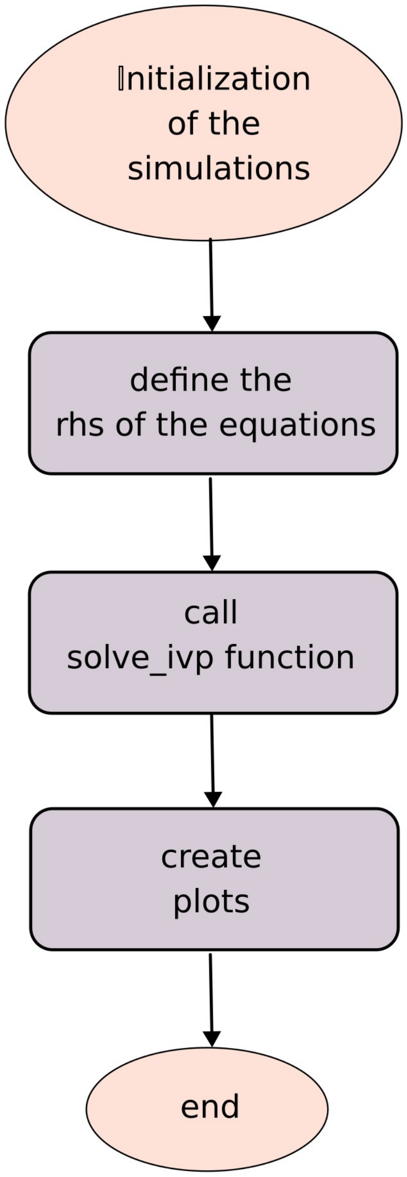

Figure S1. Flowhart of the numerical simulations.

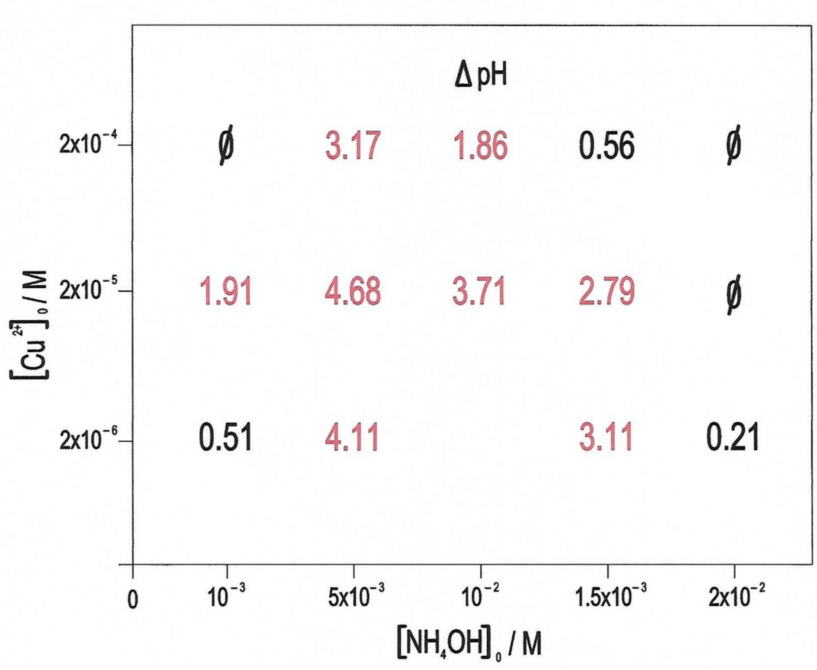

Figure S2. Amplitude of the high amplitude pH oscillations (ΔpH) in the $[Cu^{2+}]_0$ − $[NH_4OH]_0$ plane at fixed $[S_2O_8^{2-}]$ = 0.02 M, $[S_2O_3^{2-}]$ = 0.005 M, $k_0 = 2x10^{-3}$, T = 25 ºC.

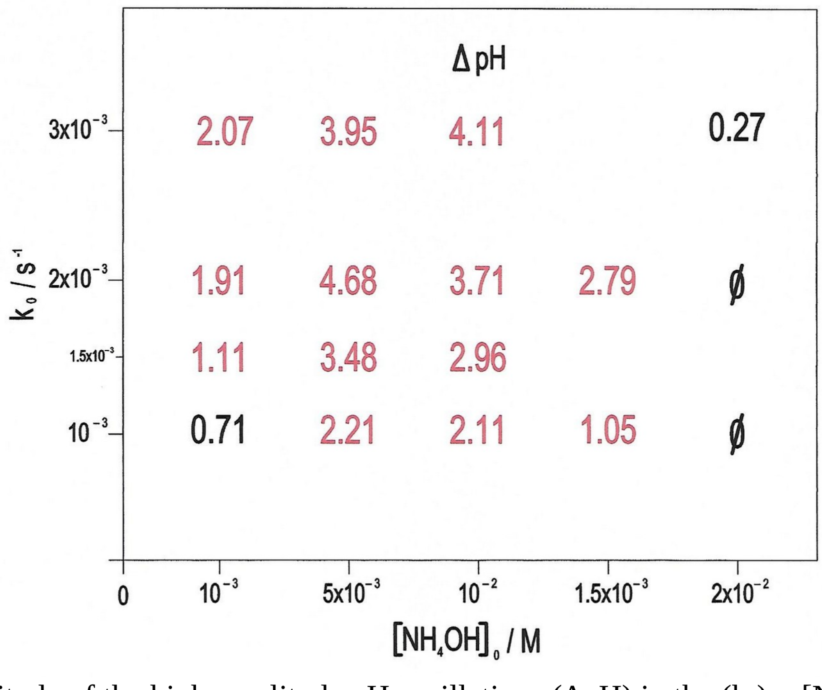

Figure S3. Amplitude of the high amplitude pH oscillations (ΔpH) in the (k_0) − $[NH_4OH]_0$ plane at fixed $[S_2O_8^{2-}]$ = 0.02 M, $[S_2O_3^{2-}]$ = 0.005 M, $[Cu^{2+}] = 2x10^{-5}$ M, T = 25 ºC.

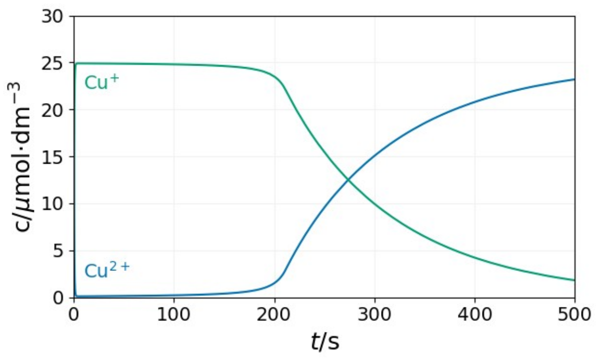

Figure S4. Simulation of the batch dynamics of the peroxodisulfate-thiosulfate-copper(II) reaction. The parameters used in the simulations are: $[S_2O_3^{2-}]_0 = 0.0025$ M, $[S_2O_8^{2-}]_0 = 0.025$ M, $[Cu^{2+}]_0 = 2.5\times10^{-5}$ M, $[H^+]_0 = 10^{-5}$ M.

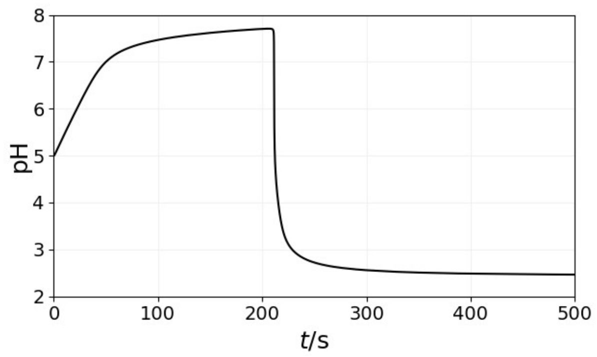

Figure S5. Simulation of the batch dynamics of the peroxodisulfate-thiosulfate-copper(II) reaction. The parameters used in the simulations are: $[S_2O_3^{2-}]_0 = 0.0025$ M, $[S_2O_8^{2-}]_0 = 0.025$ M, $[Cu^{2+}]_0 = 2.5\times10^{-5}$ M, $[H^+]_0 = 10^{-5}$ M.

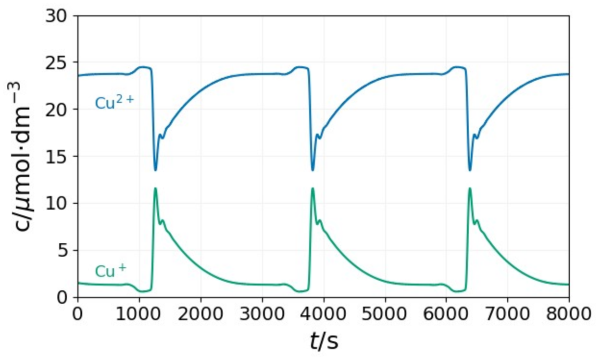

Figure S6. Simulation of the CSTR dynamics of the peroxodisulfate-thiosulfate-copper(II) reaction. The parameters used in the simulations are: $[S_2O_3^{2-}]_0 = 0.0025$ M, $[S_2O_8^{2-}]_0 = 0.025$ M, $[Cu^{2+}]_0 = 2.5\times10^{-5}$ M, $[H^+]_0 = 10^{-5}$ M, $k_0 = 1.2\times10^{-3}$ s^{-1}.

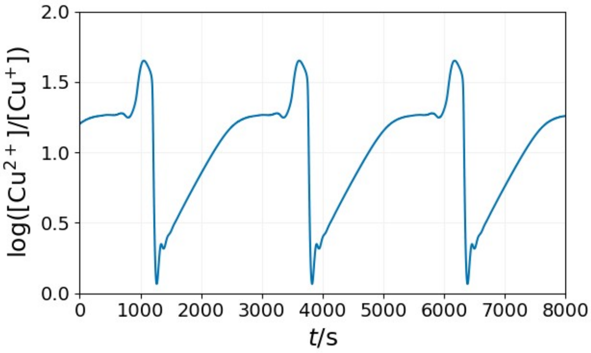

Figure S7. Simulation of the CSTR dynamics of the peroxodisulfate-thiosulfate-copper(II) reaction. The parameters used in the simulations are: $[S_2O_3^{2-}]_0 = 0.0025$ M, $[S_2O_8^{2-}]_0 = 0.025$ M, $[Cu^{2+}]_0 = 2.5\times10^{-5}$ M, $[H^+]_0 = 10^{-5}$ M, $k_0 = 1.2\times10^{-3}$ s^{-1}.

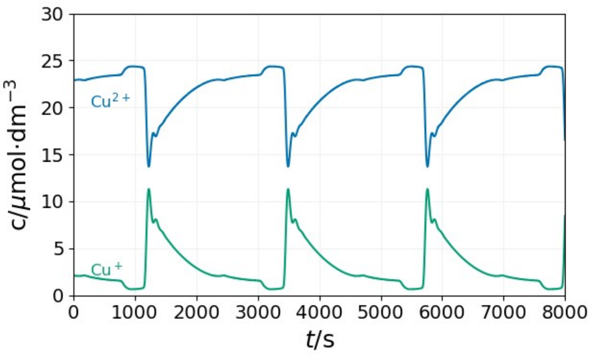

Figure S8. Simulation of the CSTR dynamics of the peroxodisulfate-thiosulfate-copper(II) reaction in presence of ammonia. The parameters used in the simulations are: $[S_2O_3^{2-}]_0 = 0.0025$ M, $[S_2O_8^{2-}]_0 = 0.025$ M, $[Cu^{2+}]_0 = 2.5\times10^{-5}$ M, $[H^+]_0 = 10^{-5}$ M, $[NH_3]_0 = 5\times10^{-4}$ M, $k_0 = 1.2\times10^{-3}$ s^{-1}.

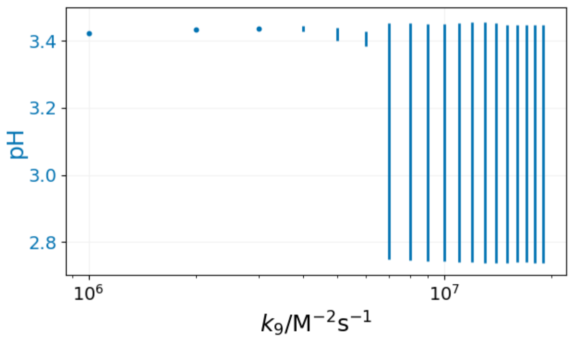

Figure S9. Simulated bifurcation diagram of the CSTR dynamics of the peroxodisulfate-thiosulfate-copper(II) reaction in absence of ammonia. The parameters used in the simulations are: $[S_2O_3^{2-}]_0 = 0.0025$ M, $[S_2O_8^{2-}]_0 = 0.025$ M, $[Cu^{2+}]_0 = 2.5\times10^{-5}$ M, $[H^+]_0 = 10^{-5}$ M, $k_0 = 1.2\times10^{-3}$ s^{-1}.

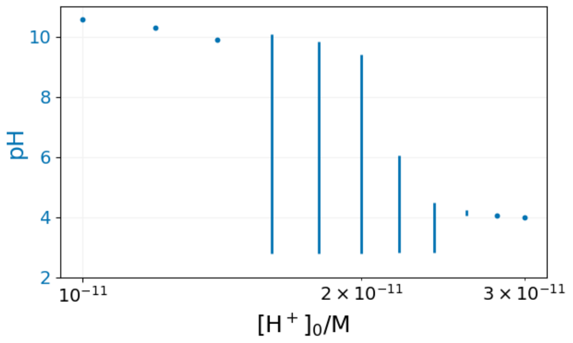

Figure S10. Simulated bifurcation diagram of the CSTR dynamics of the peroxodisulfate-thiosulfate-copper(II) reaction in absence of ammonia. The parameters used in the simulations are: $[S_2O_3^{2-}]_0 = 0.0025$ M, $[S_2O_8^{2-}]_0 = 0.025$ M, $[Cu^{2+}]_0 = 2.5\times10^{-5}$ M, $k_0 = 1.2\times10^{-3}$ s^{-1}.
